# Point-of-care ultrasound in patients with aortic dissection – two year experience at Ljubljana Emergency Medical Unit

**DOI:** 10.1186/2036-7902-4-S1-A14

**Published:** 2012-12-18

**Authors:** Hugon Možina, B Jug, M Podbregar

**Affiliations:** 1 Emergency Medical Unit, University Medical Centre Ljubljana, Zaloška 7, Ljubljana, Slovenia; 2 Dept. of Vascular Diseases, University Medical Centre Ljubljana, Slovenia; 3 Dept. of Intensive Care Medicine, University Medical Centre Ljubljana, Slovenia

## Background

Aortic dissection (AD) is associated with high morbidity and mortality; mortality rates increase by 1-2% per hour1, therefore timely diagnosis is pivotal. Point-of-care ultrasound (PoCUS) narrows the number of differentials in patients with shock, chest pain and shortness of breath2; however, reports addressing its routine use in the ER are scarce.

## Objective

Retrospective assessment of factors influencing use of PoCUS and its impact on time-to-diagnosis in patients with AD.

## Patients and methods

We reviewed medical records and charts of patients with confirmed diagnosis of acute AD between May 2010 to May 2012.

## Results

Twenty-seven patients (out of 45.630 presenting to the ER) with AD were identified (19 type A, 8 type B; 13 with typical clinical presentation). Diagnosis was confirmed with contrast enhanced CT in 25 patients, and with PoCUS (during CPR) and autopsy in two. 14 (52%) had prior PoCUS (11 confirmed, 3 supported the diagnosis). PoCUS did not affect time-to-discharge from the ER significantly (87, 60-120 vs. 120, 102-240 minutes, p=0.179). PoCUS was performed more often in unstable patients (100 vs. 38.1% stable, p=0.09) and in patients with equivocal clinical presentation (30.8% vs. 71.4% in typical presentation, p=0.038). On a multivariate model, atypical clinical presentation emerged as an indenpedent predictor of PoCUS use after adjustment for age, gender, and hemodynamic stability (p=0.047).

## Conclusion

Our findings suggest that point-of-care ultrasound is increasingly used in the initial ER management of patients with AD, especially in hemodynamically unstable patients and in patients with atypical clinical presentation.
